# Use of Telemedicine to Screen Patients in the Emergency Department: Matched Cohort Study Evaluating Efficiency and Patient Safety of Telemedicine

**DOI:** 10.2196/11233

**Published:** 2019-05-08

**Authors:** Nicholas James Rademacher, Gai Cole, Kevin J Psoter, Gabor Kelen, Jamie Wei Zhi Fan, Dennis Gordon, Junaid Razzak

**Affiliations:** 1 Department of Emergency Medicine University of Michigan Ann Arbor, MI United States; 2 The Johns Hopkins School of Medicine Johns Hopkins University Baltimore, MD United States; 3 Department of Pediatrics The Johns Hopkins School of Medicine Baltimore, MD United States; 4 Department of Emergency Medicine The Johns Hopkins School of Medicine Baltimore, MD United States; 5 Center for Population Health IT Bloomberg School of Public Health Johns Hopkins University Baltimore, MD United States

**Keywords:** telemedicine, telehealth, screening, triage, emergency medicine, emergency health services, emergency medical service, left without being seen, emergency room, emergency department, tele-medicine

## Abstract

**Background:**

Early efforts to incorporate telemedicine into Emergency Medicine focused on connecting remote treatment clinics to larger emergency departments (EDs) and providing remote consultation services to EDs with limited resources. Owing to continued ED overcrowding, some EDs have used telemedicine to increase the number of providers during surges of patient visits and offer scheduled “home” face-to-face, on-screen encounters. In this study, we used remote on-screen telemedicine providers in the “screening-in-triage” role.

**Objective:**

This study aimed to compare the efficiency and patient safety of in-person screening and telescreening.

**Methods:**

This cohort study, matched for days and proximate hours, compared the performance of real-time remote telescreening and in-person screening at a single urban academic ED over 22 weeks in the spring and summer of 2016. The study involved 337 standard screening hours and 315 telescreening hours. The primary outcome measure was patients screened per hour. Additional outcomes were rates of patients who left without being seen, rates of analgesia ordered by the screener, and proportion of patients with chest pain receiving or prescribed a standard set of tests and medications.

**Results:**

In-person screeners evaluated 1933 patients over 337 hours (5.7 patients per hour), whereas telescreeners evaluated 1497 patients over 315 hours (4.9 patients per hour; difference=0.8; 95% CI 0.5-1.2). Split analysis revealed that for the final 3 weeks of the evaluation, the patient-per-hour rate differential was neither clinically relevant nor statistically discernable (difference=0.2; 95% CI –0.7 to 1.2). There were fewer patients who left without being seen during in-person screening than during telescreening (2.6% vs 3.8%; difference=–1.2; 95% CI –2.4 to 0.0). However, compared to prior year-, date-, and time-matched data on weekdays from 1 am to 3 am, a period previously void of provider screening, telescreening decreased the rate of patients LWBS from 25.1% to 4.5% (difference=20.7%; 95% CI 10.1-31.2). Analgesia was ordered more frequently by telescreeners than by in-person screeners (51.2% vs 31.6%; difference=19.6%; 95% CI 12.1-27.1). There was no difference in standard care received by patients with chest pain between telescreening and in-person screening (29.4% vs 22.4%; difference=7.0%; 95% CI –3.4 to 17.4).

**Conclusions:**

Although the efficiency of telescreening, as measured by the rate of patients seen per hour, was lower early in the study period, telescreening achieved the same level of efficiency as in-person screening by the end of the pilot study. Adding telescreening during 1-3 am on weekdays dramatically decreased the number of patients who left without being seen compared to historic data. Telescreening was an effective and safe way for this ED to expand the hours in which patients were screened by a health care provider in triage.

## Introduction

Over nearly three decades, the volume of emergency department (ED) visits has steadily grown [1-3]. The inability to slow down utilization has resulted in continued ED crowding and considerable delays prior to ED evaluation and treatment with the associated adverse effects on patient outcomes [4-9].

One solution to expedite emergency care in the face of growing demand is to place a provider proximate to triage evaluation. Apart from fulfilling requirements of the Emergency Medicine Treatment and Labor Act, early provider evaluation assists with (1) identification of patients who may be critically ill but not yet classified as such by the triage nurse, (2) identification of patients who can be quickly discharged, (3) early initiation of treatment, and (4) reduction in the number of patients who left without being seen (LWBS) by a qualified medical provider (typically a physician, nurse practitioner, or physician assistant). ED screening is particularly important for patient safety during times of surge and during hours with reduced staffing, when patient volume and crowding outpace an ED’s ability to provide prompt evaluation [10,11].

The application of telemedicine to screening (“telescreening”) is one additional solution to address the increased ED demands. Through a real-time audio-visual interface between patients and remote care providers, telescreening optimizes providers’ time, potentially minimizes expensive staffing requirement, and may increase the pool of providers available during undesirable times due to the ability to provide care from home or other remote settings.

Telemedicine in the ED has traditionally been used to connect minor treatment clinics to larger EDs and to facilitate specialty consultation [12-19]. Additional applications, such as adding remote providers during times of patient volume surge [20], and direct-to-consumer home visits [21] have recently shown to be effective for and popular among patients.

In April 2016, our ED initiated a pilot telescreening program to expand the hours in which screening by a provider in triage took place. The objective of this evaluation was to compare the efficiency and patient safety metrics between ED remote real-time telescreening and in-person screening encounters.

## Methods

**Figure 1:**
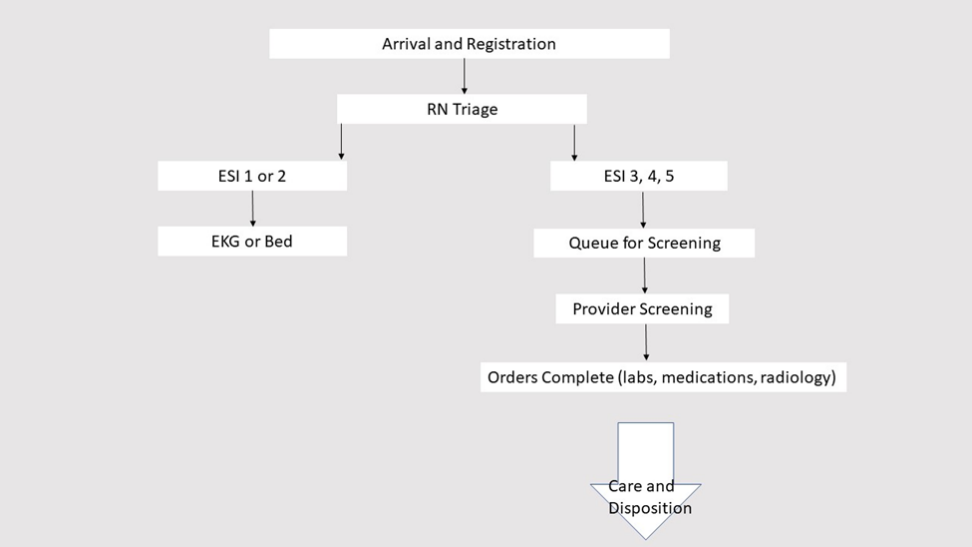
Process flow for patient intake to the emergency department. RN: registered nurse, ESI: Emergency Severity Index; EKG: electrocardiogram.

We conducted a matched cohort study to compare the performance of remote real-time telescreening (hereafter referred to as telescreening) and in-person screening at a single urban academic ED with 67,620 adult patient visits in 2016. This ED is part of a quaternary care, 900-bed, academic medical center serving a mix of predominantly inner-city, suburban, and international patients. At the time, triage was performed by registered nurses using the Emergency Severity Index (ESI) [22]. Patients with ESI levels 1 and 2 were triaged directly to an ED bed, including hallway beds, for a full evaluation and bypassed ED provider screening. Patients with ESI levels 3, 4, and 5 were briefly evaluated or “screened” by a physician, nurse practitioner, or physician assistant (Figure 1).

This analysis was conducted from April to August 2016. These months were chosen because telescreening was initiated in April 2016 with a process similar to that used in in-person screening. In early September 2016, the screening process changed, causing the screening blocks to no longer serve as suitable controls for the telescreening periods. Telemedicine was generally offered during this period, from Tuesday to Friday, 1-3 am, and on Saturdays and Sundays, 7-10 am. Additional hours were included depending on provider availability and need. This period was not previously covered by any screening activity. Telescreening was contiguous with on-site screening, that is, it naturally followed weekday on-site screening and was continued on the weekends. Although these blocks of time were not officially classified as times of “surge,” patient volume typically outpaces the capacity of the local ED system, resulting in waiting for all, but the most critically ill patients, similar to surge situations.

All adult patients during these times triaged to ESI levels 3 through 5 were offered telescreening. Since this was not an established practice, written informed consent for telescreening was obtained by certified nursing assistants (CNAs). Patients who did not consent were resigned to the usual protocol available at the time. Non-English–speaking patients and those deemed devoid of mental capacity, including those with an altered mental status, were not eligible to receive telescreening and were relegated to usual care. After registration and triage, appropriate patients proceeded to a screening (tele- or in-person screening) evaluation, which aimed at attending to the patients within 30 minutes of their arrival. The Institutional Review Board exempted this project based on its quality improvement classification. We did not charge professional fees for telemedicine screening.

Five providers from our institution—three physicians and two physician assistants—who were accustomed to in-person screening in the same ED, received technical training in the telescreening procedures as well as mock standardized patient encounters. These training sessions consisted of technical training on how to use the Clearsteth stethoscope (GlobalMedia Group, LLC, Scottsdale, AZ) and run the accompanying software (Polycom, San Jose, CA). A set of five live patient models were designed to allow providers to practice using the telemedicine equipment as well as write notes and enter orders during the exam. Training sessions were the same for each type of provider and were supervised by one of the authors of this manuscript (JR or NR) for proficiency. Screening was performed by 27 providers (physicians, nurse practitioners, or physician assistants).

We used the Globalmed (GlobalMedia Group, LLC) Clinical Access Station. The customized device utilized two-way high-quality audio and high-resolution cameras attached to a Polycom codec, with pan and zoom controlled by the telescreening provider or the CNAs trained to set up and facilitate use of the Clinical Access Station. Our Clinical Access Station had a personal computer, two monitors, a fiber optic light source, and three peripherals: a high-resolution hand-held camera, a fiber optic otoscope, and a stethoscope. Multimedia Appendix 1 shows our Clinical Access Station. The CNAs were trained in the use and placement of the peripherals to optimize information (images and auscultation) transmitted to the remote health care providers. The remote health care provider connected with the onsite system using a dual-monitor computer via Polycom for video and Clearsteth for auscultation. Multimedia Appendix 2 shows the interface from the perspective of both the patient and the remote screener.

Software was available for use on an institutional license and provided high-definition, HIPAA (Health Insurance Portability and Accountability Act)-secure, two-way communication between the remote health care provider at their homes and the exam room. All orders and telemedicine screening notes were placed in the existing electronic medical record framework (EPIC, Verona, WI).

Because the telescreening time represented incremental coverage hours, there were no time-matched historical control time periods; therefore, we matched these time periods to equivalent proximate hours to control for day of the week and ED volume. For example, if telescreening was conducted from 1 to 3 am, we matched the time with the most proximate in-person screening, which was 11 pm to 1 am. In addition, to evaluate the effect of telescreening on rates of LWBS as compared to no screening, each telescreening and in-person screening hour was matched to the corresponding day and time in the preceding year (2015). Information on individuals entering the ED during these hours was then abstracted from our EMR; this information included basic demographics, medications ordered, chief complaint, and final disposition.

Scheduled telemedicine shifts that could not be fulfilled for technical or assistant staff’s shortfalls were excluded from analysis. However, given that matching was done based on the expected telescreening shifts, the asymmetrical number of hours between the groups can be accounted for by canceled telescreening hours. The matching approach was utilized to control for day of the week and ED volume.

The primary outcome of interest was the number of patients screened per hour. Secondary outcomes of interest included LWBS rates, patients receiving analgesia (ibuprofen, acetaminophen, ketorolac, oxycodone, hydrocodone, hydromorphone, morphine, tramadol, naproxen, dicyclomine, codeine, diclofenac, fentanyl, meloxicam, or methadone), and the ordering of a chest pain bundle.

Patients who LWBS included only patients physically presenting during the evaluation period. For example, if screening occurred from 1 am to 3 am, only patients registering during those times were evaluated to determine the LWBS rates. Rates of analgesia administration were compared as a quality metric. Screeners initiated plans of care; therefore, the time for which the patients were in the waiting room was used to obtain results of laboratory and radiological tests. Similarly, screeners worked toward achieving patient comfort by ordering oral analgesia while the patients waited for a formal evaluation. As pain is one of the most common complaints of patients presenting to the ED, our ability to provide safe and effective palliation is a quality metric. Given that screeners provide basic oral analgesia to those returning to the waiting room to complete their care, it is important that telescreeners provide this care at a similar rate.

As chest pain is a common chief complaint with a high-risk profile, initiation of evaluation of patients with this presenting complaint was used to compare safety and quality between the two modes of screening. The chest pain bundle, which was considered to represent standard orders by health care providers on the research team prior to analysis, included the following items: complete blood count, comprehensive or basic metabolic panel, troponin I levels, electrocardiogram, chest x-ray, and aspirin. If components of the bundle were performed prior to evaluation by the screener, that component was counted as successfully being provided. In some cases, ordering providers placed their orders through an order set designed for patients presenting with chest pain. Manual review of charts of patients presenting with chest pain was performed by one physician-author (NR).

Immediately after a telescreening encounter, patients were given a six-question Likert scale questionnaire to complete. The focus of the questionnaire was patient satisfaction. As such, the questionnaire was not validated, and the response rate is unknown. The results from this survey are shown in Multimedia Appendix 3.

Demographic and clinical characteristics of individuals who received telescreening and in-person screening were compared using chi-square or the Fisher exact test for categorical variables and the Student *t* test for continuous variables, with unequal variances. For the subgroup of individuals presenting with chest pain and screened, receipt of a chest pain bundle was compared between the two screening modes. The mean number of individuals screened per hour, rates of LWBS, and rates of analgesia ordered were compared between telescreening and in-person screening hours in a similar manner. We compared the screening hours and proportions of patients who LWBS between 2016 and 2015 for both telescreening and in-person screening hours. The 95% CI was considered significant, and all analyses were conducted using STATA Version 14.1 (StataCorp, College Station, TX).

This trial is reported in accordance with CONSORT-EHEALTH [23].

## Results

From April to August 2016, telescreening was performed for 315 hours and in-person screening was performed for 337 proximate matched hours. During these hours, a total of 3430 individuals were screened, of which 1497 (43.64%) were telescreened and 1933 (56.36%) were screened in person. Demographics, chief complaints, and ESI level of patients who underwent telescreening were comparable to those receiving in-person screening (Table 1). Compared to patients screened in person (46.19%), a greater proportion of individuals telescreened were male (52.22%). Distribution of discharge and disposition status also differed between patients screened in person and those who were telescreened: 65.29% were discharged in the screening group (n=1262) compared to 64.19% in the telescreening group (n=961). A higher proportion of patients presenting during telescreening hours had ESI levels of 3-5 (1904/2341; 81.33%) as compared to the proportion of patients presenting during in-person screening hours (2235/2869; 77.90%; difference=3.43%; 95% CI 1.24-5.62). The total number of telescreened patients and patients screened in person was less than that of patients with ESI levels 3-5 due to the exclusion criteria applied and patient refusal for telescreening. In addition, 24.92% of the hours met our goal door-to-provider time of less than 30 minutes (77/309) for telescreening as compared to 33.23% (111/334) for in-person screening (difference=8.31%; 95% CI 1.33-15.29). The five providers performed 695, 631, 115, 32, and 24 telescreening encounters.

**Figure 2:**
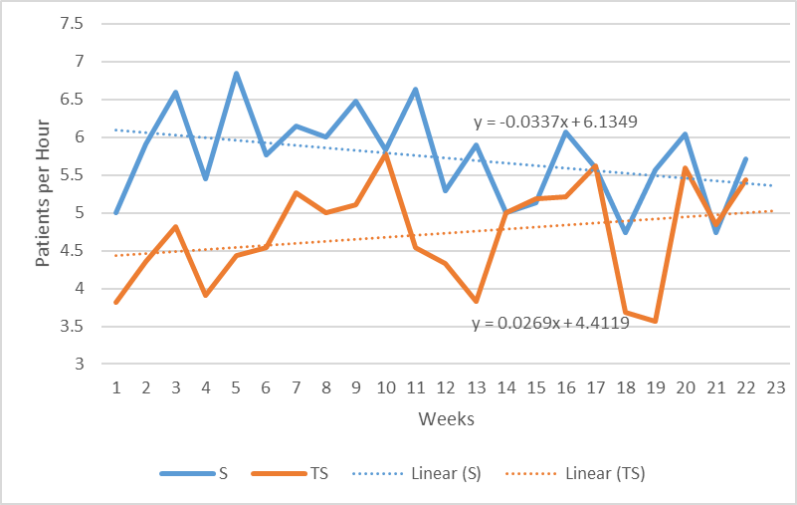
Weekly trends in patients screened per hour by in-person provider screening and remote telescreening. S: in-person screening; TS: telescreening.

On an average, 4.87 patients received telescreening per hour compared to 5.75 patients in the in-person screening group (difference=–0.87; 95% CI –1.23 to –0.51). Although a statistically significant difference was observed in the number of patients evaluated per hour in the first 3 weeks following implementation of telescreening (5.88 for in-person screening vs 4.40 for telescreening; difference=1.48; 95% CI 0.64-2.33), no differences were observed in the final 3 weeks of the study (5.52 for in-person screening vs 5.49 for telescreening; mean difference=0.03; 95% CI –0.89 to 0.94; Figure 2).

**Figure 3:**
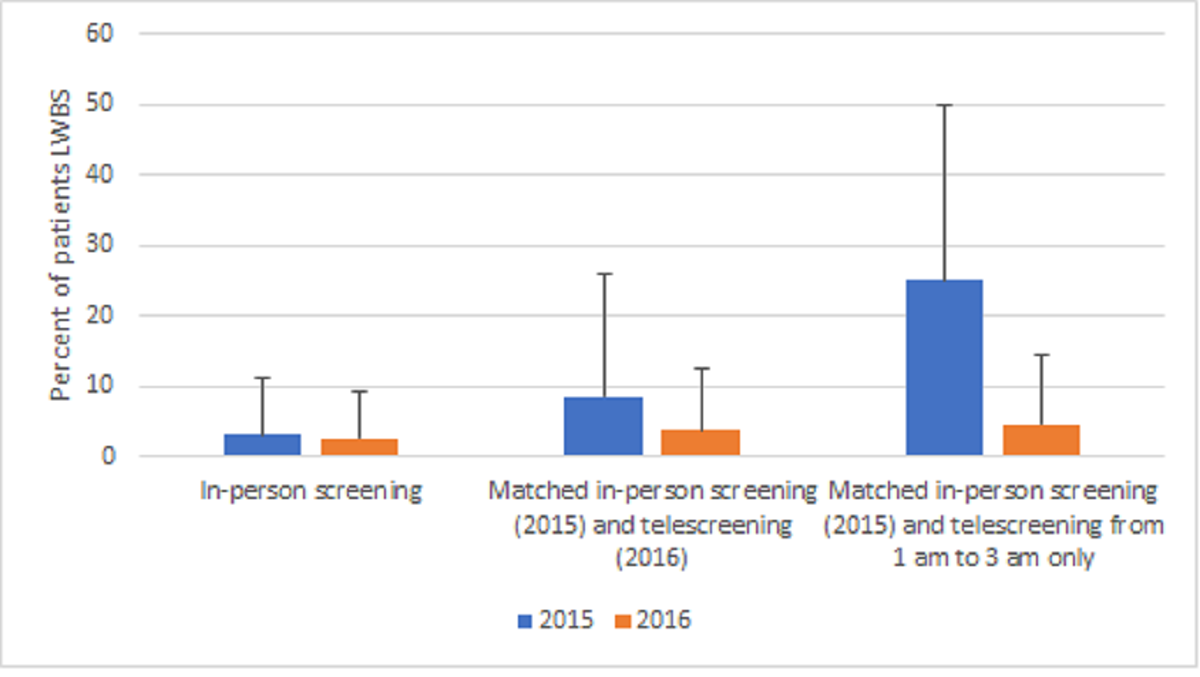
Comparison of screening modes and times between 2015 and 2016. The graph shows a comparison of patients who received in-person screening, matched in-person screening in 2015 and telescreening in 2016, and in-person provider screening (2015) and telescreening from 1 am to 3 am only (2016). LWBS: left without being seen.

The LWBS rates were higher in the telescreening group than in the in-person screening group (3.8% vs 2.6%; difference=1.2; 95% CI 0.1-1.9). However, while the LWBS rates were not different during periods of in-person screening in 2015 and 2016 (difference: 0.5; 95% CI: –0.7 to 1.6), the LWBS rates in the telescreened hours in 2016 were significantly lower than those in the matched 2015 hours (3.8% vs 8.5%; difference=–4.7; 95% CI –8.6 to –1.0). The difference from 2015 to 2016 was most pronounced in the subgroup receiving telescreening from 1 am to 3 am on weekdays. For this subgroup, the LWBS rate declined from 25.1% to 4.5% (difference=20.7; 95% CI 10.1-31.2; Figure 3).

**Table 1:** Demographic characteristics of patients in the in-person screening and telescreening groups.

Characteristic			In-person screening (n=1933)^a^	Telescreening (n=1497)^a^
**Demographics**				
	Age (years), mean (SD)		43.44 (16.8)	43.09 (15.7)
	**Gender, n (%)**			
		Male	893 (46.19)	782 (52.22)
		Female	1040 (53.80)	715 (47.76)
	**Race, n (%)**			
		White	443 (22.91)	318 (21.24)
		Black	1290 (66.74)	1071 (71.54)
		Asian	34 (1.76)	13 (0.87)
		Other	145 (7.50)	81 (5.41)
	**Ethnicity, n (%)**			
		Hispanic	95 (4.91)	59 (3.94)
		Non-Hispanic	1796 (92.91)	1409 (94.12)
**Presentation, n (%)**				
	**Disposition**			
		Admitted	227 (11.74)	124 (8.28)
		Discharged	1262 (65.29)	961 (64.19)
		Left against medical advice	18 (0.93)	22 (1.47)
		Transferred	2 (0.10)	0 (0.0)
		Eloped	21 (1.09)	29 (1.94)
		Screen and leave	300 (15.52)	288 (19.24)
		Other	103 (5.33)	73 (4.88)
	**Emergency severity index level**			
		3	1512 (78.22)	1127 (75.28)
		4	387 (20.00)	347 (23.18)
		5	20 (1.04)	19 (1.27)
	**Chief complaints**			
		Abdominal pain	201 (10.40)	162 (10.82)
		Chest pain	152 (7.86)	126 (8.42)
		Other	1580 (81.7)	1209 (80.76)

^a^Categories may not sum up to the total due to missing data.

On an average, 51.2% of telescreened patients received analgesia, compared to 31.6% of those screened in person (difference=19.6; 95% CI 12.1-27.1). Two of the providers that completed 277 of the 315 (87.9%) telescreening hours did not show a difference in the rates of ordering analgesia (Table 2). However, they ordered more analgesia per patient encounter than the group that performed the in-person screening. Although screeners ordered analgesia for 32% of the patients, the two primary telescreeners ordered 51% and 45% of that proportion when they acted as in-person screeners in a small sample of shifts prior to the implementation of telescreening.

In the subgroup of patients presenting with undifferentiated chest pain, 22.4% (34/152) who received in-person screening and 29.4% (37/126) who received telescreening had all the components of the chest pain bundle provided to them or ordered after their screening encounter *(* difference=–7.00, 95% CI –17.35 to 3.35). Analysis of individual components of the chest pain bundle revealed that aspirin administration was the only item with a statistically significant difference between the screening methods. The fact that many of the orders were likely placed as part of an order set explains some of the congruency in ordering practice. In addition, 37.3% (47/126) of the telescreened patients received aspirin and 25.0% (38/152) of the patients screened in person received aspirin (difference=12.30; 95% CI 1.41-23.19; Table 3). The results of our patient satisfaction questionnaire can be found in Multimedia Appendix 3.

**Table 2:** Rate of analgesia orders by the two primary telescreeners according to the screening method.

Primary telescreeners	Number of hours	Percentage per hour, mean (SD)		Absolute effect size (95% CI)	Relative effect size (95% CI)
		In-person screening	Telescreening		
Overall	40	48 (12)	48 (16)	–0.24 (–11.43 to 10.96)	1.00 (0.82 to 1.22)
Provider 1	26	51 (13)	44 (14)	–7.61 (–21.70 to 6.48)	0.85 (0.62 to 1.18)
Provider 2	14	45 (10)	57 (18)	11.78 (–7.19 to 30.75)	1.26 (0.91 to 1.75)

**Table 3:** Comparison of the order rates of the chest pain bundle and its components among patients in the telescreening and in-person screening groups.

Orders	In-person screening (n=152), n (%)	Telescreening (n=126), n (%)	Absolute effect size (95% CI)	Relative effect size (95% CI)
Full chest pain bundle	34 (22.4)	37 (29.4)	7.0 (–5.1 to 19.1)	31.3 (–19.9 to 115.6)
Complete blood count	136 (89.5)	115 (91.3)	1.8 (–20.7 to 24.3)	2.0 (–21.1 to 31.7)
Metabolic panel	135 (88.8)	113 (89.7)	0.9 (–21.5 to 23.2)	1 (–22.1 to 30.61)
Troponin I level	116 (76.3)	104 (82.5)	6.2 (–14.9 to 27.3)	8.1 (–17.8 to 42.2)
Electrocardiography	140 (92.1)	118 (93.7)	1.6 (–21.2 to 24.3)	1.7 (–21.1 to 30.8)
Chest radiograph	119 (77.6)	105 (83.3)	5.0 (–16.2 to 26.3)	6.44 (–18.9 to 39.6)
Aspirin administration	38 (25.0)	47 (37.3)	12.3 (0 to 25.6)	49.2 (–4.8 to 135.2)

## Discussion

### Principal Findings

This matched cohort study of our pilot telescreening program shows that telescreening can be efficiently and safely used for screening patients presenting to the ED. Although telescreening was initially less efficient than in-person screening, by the final 3 weeks of our analysis, telescreening had achieved efficiency levels similar to those of in-person screening. We included full data without a phase-in period to obtain an estimate of how long it may take for the telemedicine program to reach in-person efficiency.

Importantly, after implementation of telescreening, the LWBS rate dropped from 25.1% (in the 2015 matched weekday 1-3 am time slots) to 4.45%. Although some of these patients simply transitioned from the LWBS category to the “screened and left” category, similar to what has been reported in a recent survey [3], they were evaluated by a health care provider and often had imaging or laboratory tests drawn prior to leaving the ED. One would expect that the population that is screened and leaves is at a lower risk of adverse health outcomes than the population that simply LWBS. This issue and the health outcomes of screening, in general, are research questions worth pursuing.

The screeners’ rates of ordering analgesia were skewed by the individual practice patterns of two telescreeners who worked a majority of the telescreening hours. Additional research should be performed on this topic, especially on the breakdown of analgesic agents.

Except for aspirin administration, the chest pain bundle was completed at a similar rate between the two screening modes. This outcome suggests that telescreeners are able to set a care plan in motion for even high-risk chief complaints. However, as patients with ESI levels 1 and 2 bypassed screening and went directly to the patient care areas, the patients included in this analysis were considered to be only at moderate risk, at best, by the triage nurse. This is a necessary safeguard for a telescreening program, and this analysis does not suggest that those with undifferentiated high-risk chief complaints can safely be cared for when their vital signs or triage assessment considers them to be in danger.

An important area of further investigation is emergency patient and medicine provider satisfaction with telemedicine. Our data (Multimedia Appendix 3) broadly suggest that patients were happy with their experience with telemedicine. Few patients refused telescreening or were unsatisfied with the services; this finding is similar to those of other studies with more formal patient satisfaction surveys [21,24]

We hypothesize that demographic differences between the two groups represent subtle differences in the populations cared for at slightly different hours of the day.

Future research should focus on the use of telemedicine in other areas of emergency medicine practice, such as observation medicine and management of patient boarding in the ED. Similarly, the cost-effectiveness of telemedicine in EDs, especially if a single remote emergency provider can provide coverage to several EDs, is an area of interest. At the policy level, reimbursement of telemedicine services in the ED and ability to practice telemedicine from outside local state jurisdictions remain areas of growing discussion. As a lack of reimbursement continues to prevent wider adoption of telemedicine in the ED, payers should select measurable criteria that would lead them to begin reimbursing the costs of telemedicine, so that we can move toward those metrics.

### Limitations

First, our data are matched by date with adjacent, but not exact, time matching and therefore do not control for the time of the day variations in patient populations and presentation patterns. Second, the providers that conduct telescreening were a relatively select group motivated to carry out telescreening for various reasons. Their general comfort with the use of technology, perhaps, played a role in their willingness to participate and be early adopters of telemedicine. This study cannot estimate the efficiency or quality impact of telemedicine when applied generally to all emergency medicine providers. Moreover, the small sample of providers performing telescreening makes data on items like analgesia and orders, which are part of a chest pain bundle, susceptible to skewing according to their practice patterns. Third, we observed a significant improvement in the efficiency of telescreening as providers became more comfortable with the use of technology, achieving a comparable level of efficiency between in-person and telescreening at week 20 of the program. We did not estimate the number of hours of telescreening per provider required to reach a comparable efficiency level. Similarly, we did not test for changes in the quality-of-care indicators with time.

Fourth, the rates of LWBS were higher during telescreening than during in-person screening hours, but this is likely due to the majority of telescreening hours occurring from 1 am to 3 am, a time period with no direct comparators. The comparator was a time period of in-person screening proximate to the telescreening shift.

Finally, the patient satisfaction questionnaire was not validated prior to administration, and the response rate was unknown, as the initial purpose was to allow patients to provide immediate feedback on this pilot program. In addition, we do not have similar satisfaction data for patients being screened, which may act as a control.

### Conclusion

Telescreening is a new tool that can help EDs provide a safe and efficient alternative to in-person screening of patients while allowing a comparable level of efficiency, decreasing rates of LWBS (as compared to periods of time when screening did not previously take place), and providing greater flexibility in the provider’s schedules.

## Appendix

Multimedia Appendix 1 View of the telemedicine cart from the patient's perspective (left) and photo of the telemedicine system with labeled components (right).

Multimedia Appendix 2 Photos from the provider's perspective (left) and certified nursing assistant facilitating a visit (right).

Multimedia Appendix 3 Results of the brief patient satisfaction survey.

## Abbreviations

ED: emergency department
LWBS: left without being seen
ESI: Emergency Severity Index
CNA: certified nursing assistant
