# Supplementary figures and images for: Use of Telemedicine to Screen Patients in the Emergency Department: Matched Cohort Study Evaluating Efficiency and Patient Safety of Telemedicine
Source: JMIR Med Inform. 2019 May 8;7(2):e11233. doi: 10.2196/11233 (PMC6530260; doi:10.2196/11233)

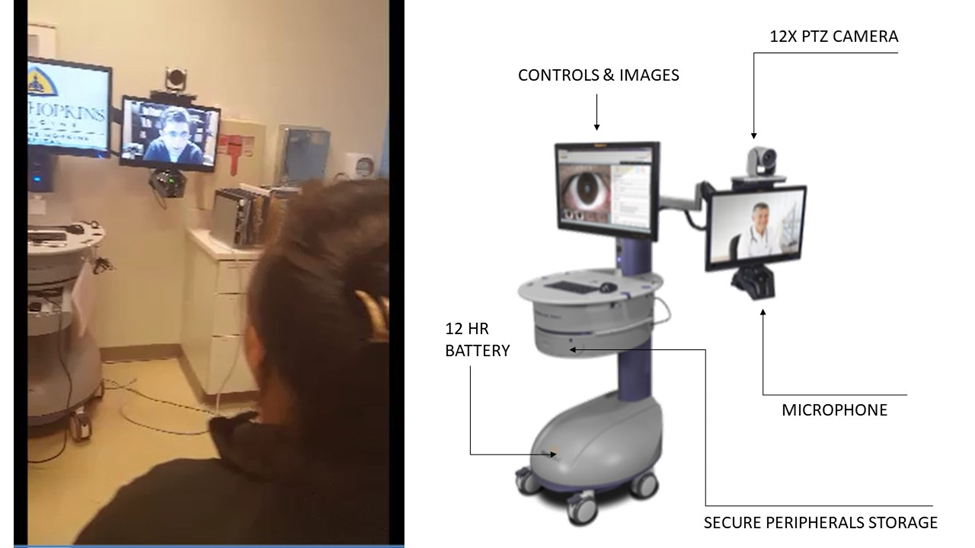

Supplement: Multimedia Appendix 1 [file medinform_v7i2e11233_app1.png]

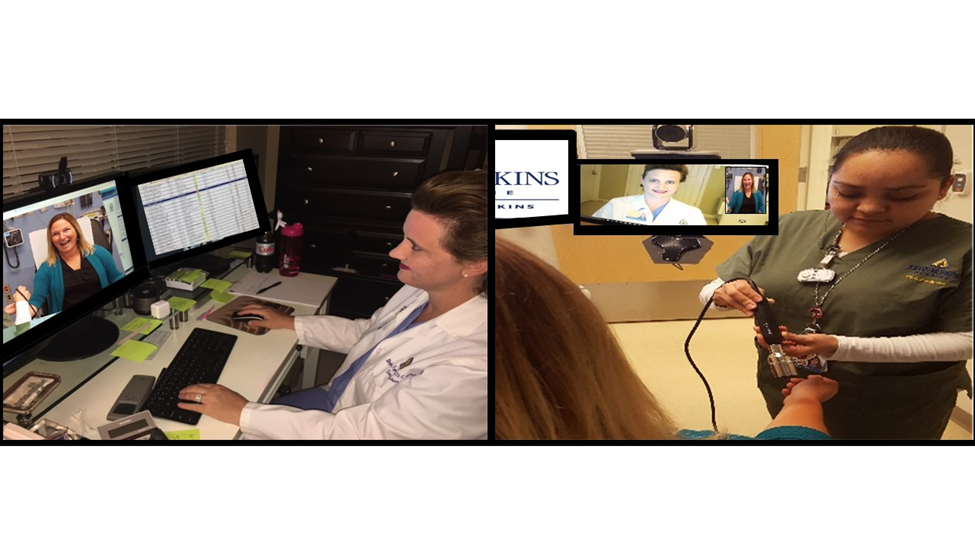

Supplement: Multimedia Appendix 2 [file medinform_v7i2e11233_app2.png]

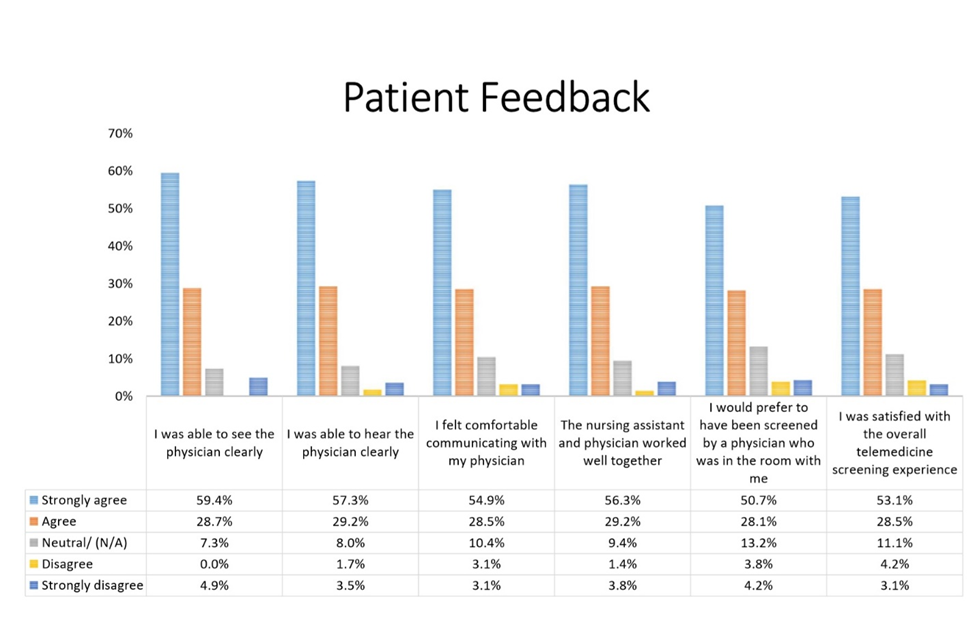

Supplement: Multimedia Appendix 3 [file medinform_v7i2e11233_app3.png]
